# French Surgery, in a Letter to Professor Gross

**Published:** 1853-10

**Authors:** Elkanah Williams

**Affiliations:** Cincinnati; Paris


ART. II.—*French Surgery*, in a letter to Professor GROSS. By ELKANAH WILLIAMS, M.D., of Cincinnati.

In my last letter I gave you an account of an operation for obliterating the vagina, resorted to in consequence of an incurable vesico-vagino-rectal fistula. By the extensive destruction of the soft parts the rectum was made to terminate directly in the vagina, and the whole mass of fecal matter and urine issued constantly and involuntarily through the vagina, giving rise to the most loathsome infirmity of which it is possible to imagine. The result of the tentative was unsuccessful, because, perhaps, of the height of the fistulous communication between the rectum and the vagina. The feces collected in the latter canal, and not being able to mount up and pass through into the rectum, forced it open, and prevented the union of the sides of its outlet. M. Maisonneuve has since divided that partition so as to make as free a connection between these two canals as possible, *near their outlet*, but the patient, not willing to submit again to the original operation, has left the hospital.

*Operation for Cataract.*—There still exists a great difference of opinion among French surgeons in regard to the superiority of the different methods of operating for cataract. M. Velpeau depresses almost exclusively, while the venerable, learned, and *eccentric* Roux is equally partial to extraction. The majority, I believe, however, are in favor of extracting as the method most generally applicable. I have seen this latter operation a number of times, and it has proven successful in most instances. Statistics prove clearly that the number of patients who recover *their sight* after extraction is greater than after depression of the lens. Both procedures are attended, sometimes, by serious inconveniences, as is every operation in surgery, however trifling in appearance. As forcibly expressed by M. Velpeau, the most insignificant cut or puncture is a door open to death. Puncture the schlerotic, depress the opaque lens, and you will frequently see intense, deep-seated inflammation arise, leading, it may be, to suppuration and complete destruction of the eye. After this simple operation, also, the patient sometimes suffers with excruciating neuralgic pains in the eye and head, which continue for months, years, and even for life. Whether this is produced by the irritation excited by the dislocated lens, or by the puncturing of the anterior part of the retina, which is inevitable in this operation, I do not know, but the fact of the occurrence of these consequences no one disputes. Serious inflammatory action follows by no means as frequently extraction as depression, but when it *does occur*, the eye is almost inevitably lost, and great deformity is the result. If the object is more especially to remove deformity, depression is certainly preferable. M. Nélaton, whom I consider the most prudent and conscientious surgeon in Paris, and who has written an admirable monograph on the comparative merits of the different modes of operating for cataract, is not very partial to either of the above operations, although he greatly prefers extraction.

In cases of *soft cataract*, when the design is to restore sight as well as remedy deformity, there is a simple procedure which, in my opinion, is preferable to all others. I refer to the method of Dr. Ware, of England, which, you know, consists in introducing a fine needle through the corner, and simply lacerating the anterior portion of the capsule of the lens, so as to subject it to the absorbing power of the aqueous humor, without dislocating it at all. Dr. W. uses a very small needle, flattened and sharp, as the cataract needle. I have seen M. Nélaton perform this operation twice — both times with success. He has modified the instrument of Dr. Ware, by slightly curving the point, so as to make it act like a hook, and prevent the cutting edge being pushed into the lens so far as to pass the capsule. He dilates the pupil with belladonna, and then introduces the needle through the cornea at about one millimètre from its edge, and simply cuts or lacerates the capsule in two or three different directions. The aqueous humor thus introduced, the lens swells, and a portion of it, in a few days, protrudes through the laceration in the capsule, and finally, after several weeks, is completely absorbed.

This operation is exceedingly simple ; is rarely, if ever, followed by any serious inflammation, and, if it fail, the eye is not *destroyed*, so that you may still have recourse to depression or extraction.

One of the cases which I saw was a man 39 years of age, and in whom the cataract was of 13 years standing. The operation required but a few seconds, produced very little pain, and was not followed by the slightest inflammatory action. This was on the 13th April. In a few days there was hernia of the lens through the capsule, and in two weeks it was so much reduced in size as to escape and fall into the anterior chamber of the eye, where it remained without any inconvenience till it was completely removed by absorption. The patient left the hospital on the 2d May.

Since that time M. Nélaton has tried the same method on a boy 8 years of age. The lens still remains in the capsule, but is being absorbed; is already divided into two fragments, between which the eye is transparent.

Recently, at the hospital of the Faculty, I have observed two cases of accidental *wounds* of the crystalline, which beautifully illustrate the steps which nature takes in destroying the lens in this operation. In one, a blacksmith, the wound was produced by a scale of iron which flew into his eye, cut through the cornea, and lacerated the capsule of the lens, which, in a few weeks, underwent the same process of hernia and complete absorption. In the other, the accident resulted from the explosion of a percussion cap. Absorption has since diminished the size of the crystalline, separated it into three portions, of which one has escaped into the anterior chamber, and the other two remain in the capsule. Between, and on each side, of these fragments the eye is transparent. It was an accidental wound of the cornea and lens, I believe, followed by the same consequences, which first suggested this operation to Dr. Ware. In instances of traumatic cataract, reported by authors, in which the lens is said to have become *again transparent*, there was, perhaps, *wound* of the *capsule*, and *complete absorption of the crystalline*. Sight is restored, not from the returning transparency, but the entire disparition of the lens. M. Nélaton thinks that this body, once opaque, never regains its transparency.

*Foreign Body in the Orbit of the Eye.*— While on the subject of the eye, allow me to give you an account of a remarkable case of foreign body in the orbit.

A man, aged 30, was struck in the eye three years ago with the *handle of an umbrella*, and rendered for some minutes, unconscious from the force of the blow. He affirms positively that he saw distinctly the shaft of the umbrella, mounted by an *ivory handle*, after the accident, and it was *not broken or shivered in the slightest degree.* He was examined by a surgeon, who found considerable contusion, and a wound at the inferior internal part of the orbit, but suspected nothing else. The acute inflammation, swelling and ecchymosis disappeared after a few days, still the wound did not completely heal, and he suffered severe pain round the orbit, and in the corresponding temple. Six months afterwards, the fistula and circumorbital pains continuing, he applied to the celebrated occulist, M. Desmarres, who sounded carefully the fistula and felt a hard substance at the bottom of it, which he supposed to be either a sequestrum of bone or a foreign body. He enlarged the fistula with a bistory, and tried to extract it, but without success. Some eighteen months after this tentative, he again applied to the same surgeon, who made a free incision in the direction of the fistula, and seized the hard body with a pair of forceps, but they slipped at each application, and brought away in the jaws a little crushed bony substance. He afterwards consulted several surgeons and physicians, who considered it *fistula lachrymalis*. On the 13th June, 1853, over three years after the accident, he entered the hospital. At first view, the man presented all the appearances of fistula lachrymalis, but, on close examination, M. Nélaton found that the lachrymal canals and nasal duct were perfectly free from obstruction. There remained a slight cicatrix and a small fistulous opening at the inferior and internal part of the orbit, *external*, however, to the lachrymal sac. On passing a probe into the fistula, it was found to extend into the orbit, backwards and inwards, and at the bottom was felt a very hard, smooth body, which gave the sensation of denuded bone. Influenced somewhat by the patient's positive declaration that he saw the ivory-mounted umbrella handle after the accident, entire, M. Nélaton concluded that it must be an *undetached* or else an *impacted* sequestrum. In order to remove it, he passed a grooved director to the bottom of the fistula, and made a free incision with a bistory. Then with a pair of forceps he seized and extracted the *entire ivory handle* of an umbrella, *two inches in length* and *three-quarters of an inch in diameter*, to the perfect astonishment of *surgeon, patient*, and all the bystanders. After the extraction of the foreign body, the patient, by blowing the nose, could make the air pass out through the opening, showing that the end of the handle had broken through the thin internal wall of the orbit into the nostril.

Previous to the operation the patient was *unable to see a particle* with the eye, although he could distinguish light from darkness. The eye-ball was a little prominent, and there was *external strabismus*, so that he could not turn the eye inwards beyond the median line. In all other directions the ball moved with perfect freedom.

The operation was performed June 13, and the slight inflammation which resulted, soon disappeared. On the 20th he could move the eye-ball freely in all directions, and began to distinguish objects. The 26th he left the hospital cured, with the *sight perfectly restored.* There are several exceedingly interesting points connected with this case —

1st. It is a warning to the surgeon not to rely with too much confidence upon the assertions and opinions of the patient, in a wound of the orbit or in any other part, and where there is room to suspect the existence of a foreign body. This man affirmed, positively and repeatedly, that he *saw* the *shaft* with its *ivory handle, after the accident*, and yet it was *all in the eye!*

A few weeks ago I saw a case at Hospital Cochin, which bears upon this same point. The man said he received a blow in the cheek from a *sabre* during the revolution of 1848, and that there had, ever since, remained a fistula. Just at the lower edge of the malar bone, and at its anterior part, was a deep depression, with a fistula in the centre, producing great deformity. At this point, also, there was firm adhesion of the soft parts of the cheek to the superior maxillary bone. In dissecting up the adhesion, M. Maisonneuve found *three flattened musket balls, of large size,* lodged against the bone. From a mistaken diagnosis, based on the patient's word, that the wound was produced by a *sabre*, an eminent surgeon had, at different times, extracted several of his teeth, on the supposition that the fistula was kept up by a carious fang.

2nd. Considering the size of the foreign body it is remarkable how little *external deformity* it produced, and the very slight derangement of the *movements* of the eyeball.

3d. The recovery of the sight after the great and protracted pressure to which the globe of the eye must have been subjected. I took the ivory handle, after the operation, and introduced it into the orbit of an adult skeleton. The posterior end *filled completely* the bottom, while the length corresponded exactly to the depth of that cavity. Although the posterior extremity of the body had broken into the nasal cavity, still there must have been, necessarily, severe compression of the retina and optic nerve, as manifested by the temporary but complete loss of vision.

*Traumatic Stricture of the Urethra, with Formation of an Immense Number of Calculi.* A man, aged 48, received a gun-shot wound in Portugal, in 1832. The ball entered at the upper and internal part of the left thigh, at its junction with the perineum, passed through the perineum, grazed the ramus of the pelvis and ischium, and escaped on the outside of the *right* nates—injuring, in its course, the urethra and rectum. When his consciousness returned, eight days after the accident, he discovered that the urine passed out at the openings of entrance and of exit of the ball, as well as by the anus ; none at all escaped by the urethra. It was only at the end of four or five months that the urine began to flow with any freedom through the natural channel, and gas from the rectum frequently issued by the same canal. About this time a splinter of bone escaped by the opening of entrance.

From thence forward the fistulous tracks ceased to give passage to the urine and gradually healed, but the stream of urine has, ever since, been small and spiral, and he has experienced pain in the perineum, and difficulty in evacuating the bladder. About four months ago his sufferings became greatly aggravated, and the obstruction to the passage of urine since becoming very great he entered the hospital on the 12th of July.

At that time the cicatrices at the points of entrance and of exit of the ball were not very manifest. There was considerable narrowing of the anus, but still the finger could be made to enter, and felt a depression, surrounded by indurated tissue, low down in the anterior wall of the rectum; and at the bottom of this depression was a little eminence which, when pressed upon, was recognized as the urethra. Coats of the rectum in other parts normal; prostate very small. The patient suffers intensely on attempting to evacuate the bladder, and cannot urinate at all when standing. He places himself on his hands and knees, and presses firmly, with his hand, the left hypogastric region, and then, only, the urine, mixed with blood and muco-pus, issues by small interrupted jets and drops. Examined with a bougie, a firm stricture was recognized at the seat of the little depression in the rectum. The smallest sized bougie was then passed into the bladder, without very great difficulty, and was felt to grate against some hard substance at the neck of that organ. The following morning all attempts to introduce the finest bougie failed, and yet he could urinate still. The pain and irritation of the bladder, with the discharge of blood and muco-pus with the urine, indicated cystitis, whether simple or complicated with a fragment of bone detached from the pelvis and lodged in the bladder, or more probably stone, which gave rise to the grating sensation, it was impossible to determine. The first indication of course was to dilate the stricture so as to admit the free exploration of the bladder. On the next day still being unable to introduce a fine bougie, M. Nélaton took a strong eight-ounce syringe, charged with *olive oil*, passed the nozzle far into the urethra, which he held firmly around it, and pushed in the injection slowly but with considerable force. Immediately after this the bougie entered without difficulty. Several times afterwards, failing to introduce the catheter, I saw him resort to the same manoeuvre with the same success. In many cases this *forced oil injection* acts admirably.

The stricture was rapidly dilated by the method of Beniqué, which consists of 36 solid metallic bougies graduated on a scale of from 1⅓ to 5 lines in diameter. Each day, at one sitting, as many of these as can be borne by the patient are introduced in rapid succession, beginning always with the highest number introduced the previous session.

On the 25th inst., a medium-sized catheter readily passed, and was felt to rub against a great number of small calculi, of which he discharged twenty-five the same evening. Since then there have escaped with the urine from fifty to sixty of these little *smooth* gravel each day, amounting in all to several hundred.

The important points in this case are, the traumatic origin of the stricture (the most intractable class of all, I believe), its long standing (twenty-one years), and the readiness with which it yielded to this method of dilatation, the immediate success of the forced oil injections in enabling the surgeon to pass the bougie, the length of time during which the urine was discharged through the rectum without inconvenience, the peculiar position assumed by the patient in order to urinate, and the immense number of calculi that formed behind the stricture.

*Stone in the Bladder—Operation through the Rectum.* A man, aged 50, while enjoying robust health, fifteen years ago, began to remark gravel in his urine. Since that period he has, from time to time, discharged immense quantities of small calculi, but continued to labor as usual. Some eighteen months ago the emission of gravel *ceased*, and symptoms of vesical irritation commenced, followed at length by several attacks of hematuria and the constant discharge of muco-pus with the urine. At the time of entering the hospital he suffered severe pain in the hypogastric region, and in the perineum, with urgent desire to pass his urine every fifteen or twenty minutes. On introducing the catheter all that part of the urethra in front of the prostate was found considerably contracted, but there was no stricture. Immediately at the commencement of the prostatic portion of this canal the sound encountered a calculus. Pushed farther on, it seemed to pass through several calculi or fragments, and was suddenly arrested, on entering the bladder by a large stone. Touched by the anus, the prostate is felt very large, projecting into the rectum, and by pressing gently upon it with the finger the calculi were made to move and rub upon each other. There was evidently great dilatation of the prostatic portion of the urethra, by a collection in it of calculi, and the intervening wall between them and the rectum was very thin. The cavity of the bladder was very much contracted, and closely applied around the large stone, which it also contained.

The projection of this prostatic pouch into the rectum, and the thinness of its walls, induced M. Nélaton to resort to an operation suggested and practiced for the first time, at least in France, by the bold surgeon of hospital Cochin, M.Maissonneuve. It is, in fact, the bilateral operation performed through the rectum in respecting the sphincter ani. The anus having been previously largely dilated (in consequence of the proverbial *amativeness* of Frenchmen, the surgeon in Paris is often saved the trouble of *this preliminary measure)*, and a grooved sound introduced into the bladder, Mons. N. inserted his finger into the rectum and, feeling for the groove in the staff, made an incision, about one centimètre in length, into the membranous portion of the urethra, just in front of the prostate.

Through this incision he passed the double lithotome caché of Dupuytren into the bladder, opened and withdrew it, so as to cut the *prostate exactly as it is divided in the bilateral operation through the perineum.* With a pair of forceps he readily removed a stone larger considerably than the largest hen's egg, and afterwards, with the fingers, extracted three bulky fragments, one of which, from its size and shape, seemed to be a scale that had separated spontaneously from the large calculus; the other two, when applied together, making a separate stone, which had been formed in the prostatic portion of the urethra. The operation was performed with much expedition, and Mons. N. remarked that he could, with great facility, explore the whole cavity of the bladder with the finger.

In some instances in which this method has been adopted there remained vesico-rectal fistula, which required a second operation. The extent and gravity of the wound is certainly much less than in the ordinary procedure of cutting through the perineum, and I imagine that in cases similar to the above it is decidedly preferable.

This patient was operated upon the 25th inst., and up to the present time no serious symptoms have arisen. He lost but very little blood in the operation, and no vessel was tied. The bowels have been evacuated several times since without difficulty; the urine passes in part through the rectum, and in part through the urethra. I shall watch the progress and final result of this case, and let you know at some future time. This morning he has no appetite, and his expression is not quite so favorable, so that it is very possible he may die, not from the grave character of the operation, but in consequence of the unwholesome atmosphere of the hospital. The fatality after operations in the Parisian hospitals is very great, notwithstanding the extreme cleanliness which prevails in all the wards.

*Hypogastric Operation for Stone.* A few months since I saw M. Nélaton perform the hypogastric operation for a stone in the bladder, which he thought was too large to be extracted through the perineum. The patient, as you are prepared to hear, died thirty-six hours after the operation, of urinary infiltration, as happens in the immense majority of such cases. M. Vidal de Cassis some years ago proposed the idea of cutting through the abdominal parieties till you arrive at the bladder, and then inserting a harpie in the wound, so as to produce inflammation and obliteration of the loose cellular tissue between the posterior surface of the pubes and this organ, and puncturing the bladder and extracting the stone some days afterwards. The liability to infiltration is certainly not so great in this procedure. It has been tried, I think, in four cases—two died and two recovered. With my present views of the matter, if I had to deal with a case of a similar kind, I should attempt to remove it through the perineum, even if I had to pass the limits of the prostate, and cut freely the bas fond of the bladder, or, what would be better, to crush the stone previous to extracting it. According to Malgaigne, the danger of *completely dividing the prostate* is not so great as has been represented.

*Stricture of the Rectum—probably Congenital.* A woman, aged about 22, entered the hospital June 4th, for an affection of the rectum. She states that she was operated upon two years ago for fistula in ano, the result of a small abscess which had formed and opened spontaneously near the verge of the anus, and from which she suffered but very little. Previously to the formation of this abscess she had suffered frequently from constipation of the bowels. Shortly after the operation for fistula, she began to experience pain in evacuating the bowels, and obstinate constipation, going sometimes nine or ten days without a stool, and was then relieved of immense quantities of fecal matter by an attack of diarrhea. The pain and difficulty in discharging the feces gradually increased till she at length suffered extreme torture, and entered the hospital for surgical aid — the calibre of the matters passed not being larger than a common goose quill. On inspection—and French surgeons are fond of *inspecting* these parts—the anus was entirely natural. The finger, however, introduced into the rectum encountered, at the height of about two inches, an obstacle — a species of diaphragm with a small opening in the center, not large enough to admit the point of the index finger. This valvular stricture was thin, supple, smooth, and easily pushed up, to a certain extent, with the finger. The walls of the rectum around it were soft, pliant, and, in all respects, normal.

M. Nélaton introduced a probe-pointed bistory, guided by the finger, passed it through the opening, and divided the stricture in several directions. Then he was able to pass the finger readily through it, but instead of finding the rectum dilated above, as he expected, it was very much contracted, and on pushing farther up he detected another stricture precisely of the same kind, which he incised in the same way. He then introduced the ordinary dilating "mèche," and continued it for several weeks. After this little operation the patient had no more difficulty in evacuating the bowels and is now cured.

In regard to the cause of this stricture I am inclined to consider it *congenital*. M. Coste, in his work on Embryology, describes the alimentary canal as originally developed in three separate parts—the esophagus and stomach forming the first, the small intestine the second, and the large intestine the third portion which terminates at the inferior part of the rectum in a "cul de sac." The anus is formed separately in the form of a sac with the closed end above. The bottoms of these blind sacs being applied together, the partition is removed by absorption, and the anus thus becomes continuous with the intestinal canal. If these intervening membranes are completely absorbed the parts are entirely natural; on the contrary, if not removed at all, there is congenital occlusion of the rectum. At all intermediate points there exist the different grades of congenital stricture.

In some places these cul de sacs are placed at some distance from each other, and are found closed at birth, and only connected by a fibrous cord, which extends between them in the situation of the absent rectum. In others they approach nearer together, each one is perforated by a small opening, and these are connected by a small hollow cylinder, so that there are two valvular strictures, and an intervening narrowed canal. This was exactly the condition of the parts in the patient above described. I see no other plausible reason to account for it, if it be not a congenital vice of formation. The very slight inflammation which preceded the small abscess and fistula from which she dates her affection, was certainly not extensive enough to have altered the structure of the rectum, and produced such a stricture. The coats of the rectum around the stricture has not that hard and fibrous feel which would certainly have been produced by an inflammation and ulceration sufficient to have led to such an organic contraction of the canal; besides, these causes do not give rise to the *kind* of stricture which existed in this case. As she had suffered from constipation for a long time previous to the appearance of the abscess and fistula in ano, it is probable that this was a mere *coïncidence*, or perhaps a *consequence*, of the stricture, instead of its cause.

There are other cases of congenital stricture of the rectum which depend probably upon an exaggerated development of the circular folds of mucous membrane in the upper part of this organ called valves of Houston. The same is true of the prominent fold containing a few muscular fibres at the point of union of the sigmoid flexure with the rectum. In 1833, Mr. O'Bierne, of Dublin, described this fold and called it the superior sphincter, and even attributed to it the most important part in the retention of the feces. He transferred the functions of the internal sphincter to his imaginary superior sphincter, and wished to make the *rectum a mere anus.* Although he exaggerated the importance of this fold, still it frequently exists in a marked degree, and may then be the seat and starting point of stricture. But if the contraction of the intestine in the above case was congenital, why is it that she did not suffer much till adult age? During early life the feces are generally thin and soft, and the patient goes often to stool; hence he does not suffer much till the age when the stricture begins to be irritated and inflamed by large masses of indurated fecal matter. There is generally, it is true, some difficulty in evacuating the bowels from early life in such cases, which becomes more troublesome as the irritating causes of indurated feces, etc., are superadded with age. In a work written on this subject, by Bouisson of Montpelier, he cites a case from Boyer of congenital stricture of the anus. The patient, from infancy, had experienced trouble in passing his stools. With age the difficulty increased, and the man was treated by different surgeons for a variety of affections. At the age of twenty he came to Paris, to consult Boyer, who diagnosed it stricture of the anus, made two lateral incisions, introduced the mèche, and the patient was soon cured. M. Bouisson reports, also, a case that occurred in his own practice, in 1847. It was a woman, 40 years of age, who complained of great pain in going to stool, and obstinate costiveness, alternating every 8 or 14 days with profuse diarrhea. She had suffered slightly from her childhood with pain in evacuating the bowels, but this had become greatly aggravated in the last ten years. The anus was natural, but at the height of two or two and a half inches was felt a thin, supple, smooth membrane, extending across the rectum, with a very small perforation in the center, the edges of which were very rigid. He operated upon the woman, and cured her in a few weeks. I am inclined to think that this variety of stricture of the rectum is not very rare, and that many cases of intractable constipation of the bowels may depend upon this same congenital deformity.

*New Method in Fractures.*—A new method of applying an immovable apparatus in cases of fracture, etc., has lately been brought into notice in Paris, and as I have seen it used several times, I consider it worth mentioning and trying. The bandage of Scultetus is prepared and wet slightly with water; a portion of plaster of Paris, baked in an oven so as to have all its moisture expelled, is then sprinkled upon each strip, which is instantly applied. In a few seconds after the bandage is thus put on, it is perfectly dry and solid. The advantage of this apparatus is, that it dries almost instantly, so that it is not necessary to keep up extension and counter-extension for any length of time, and there is not the same risk of shortening of the limb before the bandage is solidified. To prevent irritation of the skin the limb should be enveloped in a roller before the plaster bandage is applied.

*Ricord's Operation for Varicocele.*—During my attendance, for the last three months, at the visits and fascinating lectures of M. Ricord, at *l'Hopital du Midi*, I have seen him apply, a number of times, his method of ligaturing the veins in varicocele; and watched with care the progress and results. He employs two needles, one sharp and the other blunt-pointed, and two strong silk ligatures. Each ligature is doubled, so as to make a loop at one extremity, and the other, formed by the two ends of the ligature, is passed a short distance through the eye of the needle. He takes hold of the scrotum with his left hand, and carefully separates the bundle of vessels from the vas deferens, and, with the right he pierces with the sharp needle the integuments, and draws the ligature through *behind* the vessels. Next he enters the blunt needle at the *point of exit* of the first, and passes it in *front* of the veins and out at the point of *entrance* of the first.

There is then, upon each side, a *loop* and the two *ends* of the doubled ligatures ; these ends are passed through the loops and drawn in opposite directions, and the vessels are thus strangulated at once. It is entirely a subcutaneous ligature and, when drawn up, presents the appearance of a simple small seton passed through the scrotum. For the first two or three days the ends of the ligatures are only drawn sufficiently to strangulate very slightly, the veins and the parts in that time become somewhat accustomed to the foreign substance. Then by means of a small grooved apparatus, which he has made for the purpose, he tightens the ligatures from day to day, by a slight turn of a small screw.

The patient is kept quietly on his back, and at the end of three weeks, or four at most, the bundle of vessels are cut, and the ligatures then drawn readily through. There is little or no inflammation, the patient hardly suffers at all, and at the end of three weeks is well. It is the simplest operation imaginable, and, according to M. Ricord's experience in several hundred cases, when all the veins are carefully included in the ligature, *never fails* to produce a radical cure. On examining the patient three or six months after the operation you cannot tell that he has ever been operated upon. I deem it more efficacious, and infinitely less dangerous, than any of the ordinary methods.

The so-called purulent absorption and the formation of metastatic abscesses is extremely common after wounds and operations in the different hospitals of Paris. Recently, the tincture of iodine, and especially the *perchloride of iron*, have been vaunted as useful in preventing this absorption, when applied to the suppurating wound or cavity. M. Nélaton thinks that, out of hundreds of cases of this accident, he has *cured* three well-marked ones by large and repeated doses of *tincture of aconite.*

Paris, July, 1853.

